# Job Insecurity, Work-Related Flow, and Financial Anxiety in the Midst of COVID-19 Pandemic and Economic Downturn

**DOI:** 10.3389/fpsyg.2021.632265

**Published:** 2021-07-15

**Authors:** Sawzan Sadaqa Basyouni, Mogeda El Sayed El Keshky

**Affiliations:** ^1^Department of Psychology, Faculty of Education, Umm AL-Qura University, Mecca, Saudi Arabia; ^2^Department of Psychology, Faculty of Arts and Humanities, King Abdulaziz University, Jeddah, Saudi Arabia; ^3^Department of Psychology, Faculty of Arts, Assiut University, Asyut, Egypt

**Keywords:** COVID-19, job insecurity, financial anxiety, work-related flow, Saudi Arabia

## Abstract

During the COVID-19 pandemic, every domain of industry has experienced a severe economic downturn with concomitant stress throughout the economy. Employees working in government and private sectors are experiencing different psychological problems. The current study was conducted to investigate the role of work-related flow in the relationship of job insecurity with financial anxiety in the employees working in private and government sectors of Saudi Arabia. The sample comprised 1,195 employees, 886 females, and 309 males. The participants' ages ranged from 25 to 60 years. The Financial Anxiety Scale, Work-Related Flow Inventory, and Qualitative Job Insecurity Measures were found valid and reliable. Structural equation modeling was applied to test the associations. As hypothesized, the results indicated that job insecurity was positively related to financial anxiety, work-related flow was negatively associated with financial anxiety, and work-related flow mediated the relationship between job insecurity and financial anxiety. All these associations were significant regardless of gender, age, marital status, sector of employment, income, self-rated health, and COVID-19 infection status. Further research is needed to understand the impact of job insecurity on financial anxiety in-depth through the paths of work-related flow, especially in the midst of COVID-19.

## Introduction

The current situation of the novel coronavirus 2019 (COVID-19) pandemic has increased levels of anxiety and depression in individuals (U.S. Census Bureau, 2020). The emergence of anxiety has been shown to be due to the pandemic's negative impacts on the economy and workforce. Concerning the transmission of COVID-19, some recommendations are given by different health organizations regarding social distancing to reduce the spread of virus, which has directly or indirectly affected businesses. These health organizations recommended closing unnecessary businesses. Even developed countries have faced economic downturn, i.e., the U.S.A. employment rate fell enormously, and over 41 million Americans became unemployed between February and May 2020 (Congressional Budget Office, 2020). Likewise, Saudi Arabia faced problems in economic productivity; the unemployment rate hit 15.4% by September 2020 (Barbuscia, 2020).

When COVID-19 was declared a pandemic, there was a significant fall in economic growth globally. Likewise, unemployment in the U.S.A. increased by 1.4 million people (U.S. Bureau of Labor Statistics, 2020). A further 33% of Americans reported that they had lost their jobs due to COVID-19 (Forbes and Krueger, 2019). The records reveal that by mid-April in 2020, the unemployment rate was significantly greater, the highest since the Great Depression (U.S. Bureau of Labor Statistics, 2020). So, due to this unfamiliar and unprecedented pandemic, the sharp declines in economic activity and employment highlight the likelihood of significant job insecurity and financial anxiety. These may have contributed to the increased anxiety in young, employed people, and even affected their efficiency in and pleasure at their work tasks/activities. Forbes and Krueger (2019) report that individual motivation toward work tasks/activities has decreased. Because COVID-19 has become prevalent everywhere and economic crises have arisen, it is necessary to study the impacts of COVID-19 on individual mental health. With the sharp economic decline, employees can experience job insecurity and can be troubled about their finances (U.S. Census Bureau, 2020).

Some studies have claimed that perceptions of job insecurity may have impact on financial anxiety of employees. Choi et al. (2020) investigated the links between job insecurity and financial stress and reported that job insecurity was related to financial stress through financial well-being. In the context of corona pandemic, another study found that job insecurity due to COVID was associated with psychological well-being through financial stress (Sarwar et al., 2021). Moreover, job insecurity was found to impact on mental health of employees. Wilson et al. (2020) found that job insecurity due to COVID increased depressive symptoms among employees. Similarly, it was revealed in Europe that increased job insecurity was related to lower vitality (Rajani et al., 2016).

The positive psychology field has paid attention on what makes employees flourish and major concepts in positive organizational behavior are engagement and flow (Bakker and Schaufeli, 2008). These positive concepts have been found to broaden employees' behavioral repertoire, resources, and skills that make them more resilient when coping with adversity (Fredrickson, 1998). Despite these positive benefits of work-related flow for employees in challenging times, no studies have investigated how work-related flow might impact well-being of employees in the midst of COVID-19. Wang et al. (2015) explored the factors affecting employee job performance. They examined the relationship between job insecurity and employee performance, as employee performance depends on work-related flow. Their results indicated that low levels of performance were present among employees who had insecure jobs. As a result, this study deals with the relationship between job insecurity and financial anxiety and aims to discover the mediating effect of work-related flow.

## Literature Review

### Job Insecurity

Job insecurity was first described in the 1980s. A person's perception was related to a sense of powerlessness regarding their threatened job (Greenhalgh and Rosenblatt, 1984). Quantitatively, job insecurity is perceived as a threat for a job as a whole (De Witte, 1999; Sverke and Hellgren, 2002), whereas qualitatively it is considered as a perceptual threat to the job's domain. Specifically, job insecurity as a qualitative experience is denoted as “threats of impaired quality in the employment relationship” (Hellgren et al., 1999). Job insecurity has various key elements such as the experience of a perceived threat, and emphasis on one's perception, and feeling that one's job conditions and security are at risk. However, job insecurity does not include expectations regarding security and involuntary threats. This distinguishes job insecurity from potential antecedents, consequences, moderators, and criticism considered by many authors (Probst, 2003; Elst et al., 2012). This construct comprises a particular continuum extending from insecure to secure where employees claim to experience job security in conditions of unthreatened, perceived continuity, and balance between a job's demands and skills. This continuum explains that job insecurity is a global construct; it does not cover only quantitative or qualitative components.

This construct of the job insecurity continuum highlights the concept of job insecurity, so it can be understood. Firstly, job insecurity is about an employee's subjective experience (De Witte, 1999). This experience comprises perceptions, although coherency is present with most conceptualizations. Job insecurity contrasts with job positions that are objectively insecure, such as jobs for temporary workers, and those subject to objective organizational circumstance such as layoffs. When considering the subjective nature of job insecurity, it is noteworthy that two employees experiencing the same objective situation may perceive different levels of job insecurity (Vuuren et al., 1991). Secondly, the notion of threat signifies job insecurity as a future-focused experience. Job insecurity denotes that an event is forecast, specifically one which can result in loss and which might occur at any time in the future. Hence, not all job-related events develop job insecurity, but only those that involve the constituents of potential harm or loss (Folkman and Lazarus, 1985; Boswell et al., 2014), because threats are considered as perceptual, whereas job insecurity involves uncertainty (De Witte, 1999; Sverke et al., 2002; Probst, 2003). Hence, job insecurity explains the intricacies of individuals' perception and their responding relationship to elements of job loss (Greenhalgh and Rosenblatt, 1984) in contradiction to the actual job or job loss elements. Finally, the job's stability and continuity are threatened by job insecurity (De Witte, 1999; Probst, 2003). This differentiates job insecurity from related other constructs, like employability, which considers an employee's perceived ability to get a new job due to their having skills related to job demands and knowledge regarding the market (De Cuyper et al., 2008).

When unemployment figures rise in countries for periods, job insecurity is considered to be the greatest fear among workers (Keim et al., 2014) and a stressor for workers in their working environment (Greenhalgh and Rosenblatt, 1984). And as job insecurity creates stress in employees, the examination of it reveals negative consequences for workers' well-being and mental health (Cheng and Chan, 2000; De Witte et al., 2016; Lee et al., 2018; Llosa et al., 2018), and significant links with depressive disorders (Blom et al., 2015; Kim et al., 2017) and anxiety too (Boya et al., 2008).

### Work-Related Flow

When an individual completely concentrates on a task/activity, they start experiencing what is called “flow,” i.e., absorption in the present time, reaches a peak where the individual becomes unaware of their personal needs and forgets their surrounding environment. Mihali Csikszentmihalyi first recognized this construct during the 1970s (Chen, 2007). The state of flow occurs within a specific zone (Chen, 2007). According to Nakamura and Csikszentmihalyi (2002), the state of flow can even bring happiness to a person's life because its effect makes them aware of a previously unknown route to happiness. A person must know about the necessary conditions for achieving the state of psychological flow, so they can successfully create the conditions for it. This construct has evolved through several refinements related to real-world states and their dynamics, and from there, the flow concept has become embedded.

A wide range of work is included within Maslow and Rogers' humanistic perspectives (McAdams, 1990). Later, Privette (1983) introduced the concept of peak performance. A performance was counted as such an episode if superior human functioning emerged within it. Further empirical literature has found that work-related flow is part of intrinsic motivation and what an individual is interested in Deci and Ryan (1985) and Renninger et al. (1992).

Three significant elements of flow are common to definitions of flow in any sort of activity: absorption, immersion in task/activity, intrinsic motivation, and enjoyment. In flow research, these three elements are considered as core components (Csikszentmihalyi and Csikszentmihalyi, 1988; Csikszentmihalyi, 1997). According to Csikszentmihalyi and Csikszentmihalyi (1988), work-related flow refers to those experiences in which an individual becomes fully engaged in a controllable situation, which involves challenging or difficult tasks/activities requiring some skill, and for which the individual is fully motivated and focused on their intrinsic factors. Self-awareness vanishes when a person is involved in such an intrinsically motivated task, and surprisingly the sense of self emerges and increases when the task is complete. During work-related flow experiences, an individual's time perception alters. The person realizes that hours seem like minutes, and when an individual does not achieve work-related flow, the opposite perceptual effects can occur (Carr, 2004).

Massimini and Carli (1988) suggested that experiencing flow requires unification between a challenging task and the required skills above a critical threshold. To remain in the flow, it is necessary to increase the task/activity's complexity by creating new challenges and skills (Csikszentmihalyi and Csikszentmihalyi, 1988). Employee capabilities and competencies should be matched with their job responsibilities, so they are likely to engage in work-related flow for a longer period (Lu, 1999). Larson (1988) claimed it is necessary to continue balancing challenging tasks/activities and competencies to an increasingly complex level as that develops growth and the discovery of personal capabilities. Work-related flow is a peak experience that helps the person to fulfill their potential (Percival et al., 2003).

When employees apply this flow state in their work, it can be considered as a short peak experience at work, that comprises absorption, work enjoyment, and an intrinsic state of work motivation (Bakker et al., 2005). Absorption has been explored as a state in which employees have full concentration on their work-related tasks/activities, and simultaneously experience short-term peak experiences. Many employees report that when they do their work in a state of flow, their time at work passes quickly, they lose interest in their surroundings and become involved in their tasks/activities (Csikszentmihalyi, 1990). Those employees who do their work with pleasure, and experience enjoyment in doing it, make positive judgments and decisions regarding their job situations and career (Veenhoven, 1984). Basically, their enjoyment and pleasurable states are consequences of affective and cognitive evaluations of work-flow experiences (Diener and Diener, 1996; Diener, 2000). Eventually, researchers discussed employee intrinsic motivation as the performance in certain work situations/job activities which resulted in experiences of pleasure, enjoyment, and satisfaction in the task/activity (Deci and Ryan, 1985). Employees who have intrinsic motivation toward their work experience interest and flow in their work (Harackiewicz and Elliot, 1998). Employees who work because of their intrinsic motivation in their work/tasks, mostly continue working because they are fascinated their office tasks/activities.

Different theories found in the existing literature suggest that the individual's task/activity should be neither too challenging nor too easy (Nakamura and Csikszentmihalyi, 2002). Simple tasks/activities do not create engrossment, and the individual is unable to achieve the work-related state of flow. When a person has a difficult task/activity, they will probably face problems in maintaining attention. This further affects the individual's attention and motivation, and consequently, they will have difficulty, discomfort, and lack of ease in handling the job and will not experience work-related flow (Chen, 2007). Csikszentmihalyi's (1997) experience in sampling studies has shown that people more often experience flow during their work than during their free time. This means that one has to invest time and energy to experience flow. Researchers generally agree that the occurrence of flow is most likely when people perceive a balance between the challenge of a situation and their skills in dealing with this challenge (e.g., Massimini and Carli, 1988; Csikszentmihalyi, 1990; Clarke and Haworth, 1994; Ellis et al., 1994).

Rogers (1980) introduced the construct of the “fully functioning person,” which seems to link with the concept of the autotelic personality (Csikszentmihalyi, 1990). According to Csikszentmihalyi (1997), an individual's personality type suggests the likelihood of their flow state. Autotelic personality-type individuals tend to maintain flow, because the autotelic personality has traits related to an internal drive and exhibits elements related to purpose and curiousness. Asakawa (2004) claims that individuals having autotelic personalities have more tendency to experience the flow state more easily than other types.

### Financial Anxiety

Financial anxiety is a recently emerging topic for researchers in the domains of financial planning, financial therapy, and financial counseling. The term “financial anxiety” refers to anxious feelings or specific worry due to a disturbance of one's financial situation (Archuleta et al., 2013). A few researchers have formalized financial anxiety by adapting criteria about anxiousness, irritability, difficulty in controlling worry, sleeping problems, concentration issues at work or school, muscle spasm or tension, and fatigue that are outlined in the Diagnostic and Statistical Manual of Mental Disorders-IV-TR for the Generalized Anxiety Disorder (American Psychiatric Association, 2000). These criteria also apply to an individual's financial situation (Archuleta et al., 2013). Financial anxiety has been considered as a syndrome consisting of psychosocial factors, the presence of which causes an individual to feel uneasy and stressed toward their finances. In the administration of personal finances, such an individual demonstrates unhealthy attitudes (Burchell, 2003).

The American Psychological Association (2015) investigated stress in the American population. Their results indicated that a majority of Americans had some degree of anxiety-related issues. According to the survey conducted in the U.S.A., the most common source of anxiety was money, followed by work and the economy. The survey results showed that people with good jobs were better at managing their financial anxiety. Approximately 25% of respondents reported that they had experienced extreme stress about money. Some participants even reported putting their medical care demands on hold due to financial concerns or money issues (American Psychological Association, 2015). Management of financial anxiety was also explored. A significant number of people reported that they tried to manage their financial anxiety by reading some books, or spending time socializing with others, their friends and family, walking, or exercising. Different research studies have been carried out, in which the overall construct of financial anxiety was measured by using subjective evaluations (Archuleta et al., 2013).

Due to job insecurity, various individuals experience financial anxiety and have deep concerns because of COVID1-19's impacts. Keeter's (2020) research shows that most employees have experienced pay cuts and that nearly 49% of Americans report that the pandemic is a major threat to their finances. Of the respondents reporting much anxiety about their finances, 57% reported medium to high levels of psychological issues and distress, anxiety, and depression.

Dijkstra-Kersten et al. (2015) indicated that financial stress has been accompanied by anxiety and depressive disorder, even for managing the income for household tasks/activities. Researchers have further argued that lifestyle adjustment problems deriving from financial problems are significantly linked to psychological well-being (Marjanovic et al., 2015; Fiksenbaum et al., 2017). Fiksenbaum et al. (2017) found that greater economic hardship can be experienced as job insecurity associated with increased anxiety regarding finances. At present, many individuals experience job insecurity due to the COVID-19 pandemic's negative consequences. They report financial anxiety and concerns about finances too. In the current prevalent situation, the huge unemployment rate and many closed businesses have raised employee job insecurity, and their financial anxiety seems to be significantly higher.

The previous literature suggests that job insecurity and financial anxiety are significantly linked; job insecurity and work-related flow have also been examined. No study indicates the relationship between job insecurity, work-related flow, and financial anxiety. This current study will focus on the predictive effect of work-related flow on job insecurity and financial anxiety and also examine the relationship between those variables.

Having job insecurity is linked with inefficient wage models. Employees have more chance of being dismissed in insecure jobs. Consequently, they are less motivated in their work, and their work-related flow decreases. The literature suggests that insecure employees regard their jobs are less productive than secure employees. Those employees who retain their jobs also experience job insecurity; they experience more than relief. In the U.S.A., Doherty and Horsted (1995) surveyed 170 employees working in financial services when many major employers were downsizing their employees with involuntary redundancy programs. They found increased levels of stress, anger, skepticism and bitterness, and decreased levels of work-related flow, motivation, morale, confidence, and loyalty to their organizations. Hallier and Lyon (1996) studied managers facing the threat of redundancy. Forty-two employees were selected for qualitative interviews. Five semi-structured interviews were carried out with each employee over 12 months. The initial interviews were done when the employees were informed about their redundancy. They experience widespread shock, despite being aware of the redundancy program. Feelings of bitterness, low work-related flow, and decreased motivation were found, and they had less trust in the employer.

**Figure 1:**
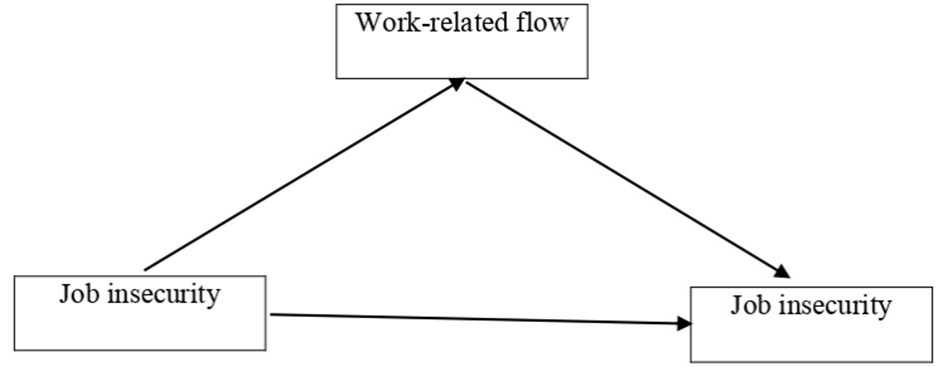
Conceptual model. Covariates: gender, age, marital status, income, SRH, and COVID infection status.

The outbreak of COVID-19 pandemic has led to loss of many jobs and this has increased job insecurity among employees. In turn, this might have impacted the work-related flow and financial anxiety of employees. This theoretical framework is displayed in Figure 1.

With regard to the theoretical and empirical evidence, the following hypothesis were tested:

*Hypothesis 1: Job insecurity will be positively associated to financial anxiety*

*Hypothesis 2: Work-related flow will be negatively associated with financial anxiety*

*Hypothesis 3: The association between job insecurity and financial anxiety will be mediated by work-related flow*

## Method

### Study Design and Sample

Different quantitative research-based techniques were used in the current study, which was conducted in a correlational structure. There were 1,195 participants involved in the study. The research was conducted using a purposive probability sampling technique. Employees of government and private sectors working in Saudi Arabia were selected as research participants. The participant age range was 25–60 years (*M* = 40.2, *SD* = 9.35), with more females (74.1%) than males. Regarding age, the modal category was middle-aged between 36 and 45 years (42.7%), and for the sector, the majority worked in the government (84.5%).

### Measures

#### Demographic Form

This form included the personal question for the study sample. The questions related to the participant's age, gender, education, employment status, and employment sector.

#### Financial Anxiety Scale

Financial anxiety was assessed by using the Financial Anxiety Scale (FAS). This scale was developed by Archuleta et al. (2013) by adapting Generalized Anxiety Disorder diagnostic criteria set forth by DSM-IV-TR (American Psychiatric Association, 2000) to a financial situation. Generalized Anxiety Disorder is characterized by excessive anxiety or worry that occurs for 6 months or longer about events or activities. This scale comprises seven items on a 7-point Likert-type scale, ranging from 1 (never) to 7 (always). Factor loadings for the scale achieved 0.72 and above, which showed its good construct validity. Internal reliability, using Cronbach's alpha, was also found to be high (α = 0.94).

#### Work-Related Flow Inventory

This inventory was developed by Bakker (2008) and consists of three factors: absorption, work enjoyment, and intrinsic motivation. The inventory comprises 13 items using a 7-point Likert-type scale, ranging from 1 (never) to 7 (always). Internal consistency of scale ranged between 0.96 and 0.75. The sample's test-retest reliability was found to be good, with stability coefficients of 0.75.

#### Qualitative Job Insecurity Measure

This scale was developed by Blotenberg and Richter (2020) and comprises 11 items and a 5-point Likert-type scale. The responses range from 1 (totally disagree) to 5 (totally agree). Internal reliability of the items was excellent and had Cronbach's α of 0.94.

### Procedures

Before the data collection, the researcher mailed the authors of the scales used in the present study to get permission for their use. Then the researcher translated the scales into Arabic and later invited employees by email and social media to participate in an online questionnaire. The invitation informed the employees about the study's aim and stated that their participation was voluntary and that they could end their participation at any time. The researcher also contacted the relevant public and private employer sectors seeking formal permission from their heads. Informed consent was obtained from the employee participants, and the researcher explained the study's aim and purpose. Their confidentiality and anonymity were ensured. They were instructed to be honest while responding to the items of the scales. Permissions to conduct this study were obtained from the Umm Al-Qura University in Saudi Arabia. In order to ensure the accuracy and originality of items, translation and back translation methods were employed with three experts in the field.

## Results

This study sought to investigate the relationship between job insecurity (JINS), work-related flow (WOLF), and financial anxiety (FAS). To achieve this, the data was collected using a Likert-scale-based structured questionnaire, and a total of 1,195 questionnaires were collected. Using G^*^Power, this sample size corresponded to a sampling power of 0.982, which according to Wywial (2015) and Kyriazos (2018) was more than the minimum prescribed power of 0.80. Alternatively, using the Kaiser criterion of 1 item: 15 observations, the minimum required sample for 31 items (JI−11; FA−7, and WOLF−13) was 465 (Oakshott, 2012). In this regard, it can be confirmed that the data collected was a good sample. The R statistical software (Ihaka and Gentleman, 1996) was used to analyze the data, and several statistical techniques were applied. Construct validity and reliability were done first, followed by the testing of the hypotheses.

### Reliability and Validity Testing

This study investigated three broad constructs, that is Job Insecurity (JINS), Financial Anxiety (FAS), and the Work-Related Flow (WOLF). WOLF was a latent variable measured by three sub-constructs, namely, absorption (ABS), work enjoyment (WE), and intrinsic work motivation (IWM). Nevertheless, prior to their use in testing the study hypotheses, construct reliability, and construct validity were tested as prescribed by Blunch (2016) and Harrington (2017). The Cronbach alpha was used to test for reliability. According to Field (2018), the minimum acceptable threshold for reliability considered is 0.70. The lowest alpha was for absorption (αABS = 0.745), and since this alpha and the others were all >0.70, which meant that the constructs were internally consistent and reliable. The results are summarized in Table 1.

**Table 1:** Construct reliability and validity.

						**HTMT**				
	**Items**	**Alpha**	**AVE**	**MaxR**	**MSV**	**JINS**	**FAS**	**IWM**	**WE**	**ABS**
JINS	13	0.908	0.893	0.989	0.444	1				
FAS	7	0.956	0.902	0.985	0.141	0.062^*^	1			
IWM	5	0.824	0.911	0.982	0.303	0.295^**^	0.376^**^	1		
WE	4	0.836	0.929	0.982	0.339	0.532^**^	0.082^**^	0.551^**^	1	
ABS	4	0.745	0.902	0.975	0.444	0.666^**^	0.100^**^	0.492^**^	0.582^**^	1

** *p < 0.01 and*

* *p < 0.05*.

Construct validity was tested using both convergent validity and discriminant validity. The Average Variance Explained (AVE) measured the convergent validity, the minimum threshold of which is 0.60 (Harrington, 2017). Since none of the construct AVEs were <0.60, the minimum being 0.893, convergent validity was not violated. For discriminant validity, the heterotrait-monotrait (HTMT) ratio was computed, and according to Beaujean (2014), its maximum tolerable threshold was 0.85. None of the constructs had an HTMT >0.85, and, therefore, discriminant validity was not violated. Overall, the constructs used in this study were confirmed to be both reliable and valid.

### Descriptive Statistics and Testing of the Hypotheses

The descriptive statistics and the mean score of financial anxiety per category of the sample are summarized in Table 2.

**Table 2:** Socio-demographic characteristics of the sample and mean of FAS per category.

**Variables**	***n***	**%**	**Mean (*SD*)**	**Mean (*SD*) of FAS**	***p***
Financial anxiety	1,195		21.8 (13.3)		
Job insecurity	1,195		26.05 (11.1)		
Work related flow	1,195		57.1 (16.6)		
Age	1,195		40.2 (9.35)		
Gender					0.03
Male	309	25.9		21.38 (13.2)	
Female	886	74.1		23.24 (13.2)	
Marital status					0.14
Not married	260	21.76		20.8 (12.8)	
Married	935	78.24		22.1 (13.4)	
Sector					0.00
Government	1,010	84.5		21 (13)	
Private	185	15.5		26.4 (13.6)	
Income					0.00
<5000RS	131	11		30 (13.8)	
5000– <9000SR	257	21.5		23.4 (13)	
9000– <13000SR	311	26		21.6 (13.2)	
9000– <13000SR	496	41.5		19 (12.2)	
Self-rated health					0.00
Weak	56	4.7		34.6 (13.1)	
Average	167	14		27.9 (13.5)	
Good	259	21.7		23.1 (12.1)	
Very good	383	32		19.7 (12.7)	
Excellent	330	27.6		18 (12.2)	
COVID infection status					0.93
No	1,028	86		21.8 (13)	
Yes	167	14		21.7 (14.6)	

The mean score for job insecurity was 26.05 (*SD* = 11.1), for financial anxiety the average score was 21.8 (*SD* = 13.3), and for work-related flow it was 57.1 (*SD* = 16.6). Most of respondents were females (74.1%), married (78.2), working in government sector (84.5%), earing between 9000 and 13000SR (41.5%), with very good health (32%), and had no COVID infections (86%).

In relation to the correlations, the results are presented in Table 3. There was a weak negative correlation between JINS and work-related flow, *r*_(1, 195)_ = −0.300; *p* < 0.05. There was also a weak negative correlation between FAS and work-related flow, *r*_(1, 195)_ = −0.227; *p* < 0.05. However, the correlation between JINS and FAS was moderate and positive, *r*_(1, 195)_ = 0.437; *p* < 0.05. Additionally, a higher level of FAS was associated with a higher level of JINS.

**Table 3:** Product-moment correlation coefficient.

	**Skew**	**Kurt**	**JINS**	**FAS**	**ABS**	**WE**	**IWN**	**WOLF**
JINS	0.493	−0.527	1					
FAS	0.585	−0.881	0.437^**^	1				
IWM	0.139	−0.757	−0.248^**^	−0.199^**^	1			
WE	−0.135	−0.945	−0.307^**^	−0.222^**^	0.754^**^	1		
ABS	−0.027	−0.904	−0.253^**^	−0.191^**^	0.626^**^	0.779^**^	1	
WOLF	−0.030	−0.814	−0.300^**^	−0.227^**^	0.880^**^	0.941^**^	0.884^**^	1

** *p < 0.01*.

**Figure 2:**
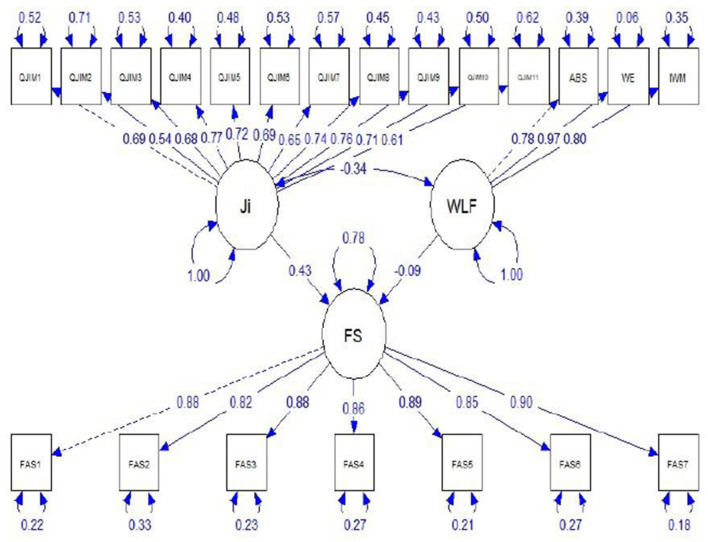
Structural equation model predicting financial anxiety.

Structural equation modeling was carried out because of the latent nature of the constructs. This SEM analysis was performed with the lavaan package (Rosseel, 2012). The results of the structural equation model are presented in Table 4 and are displayed visually in Figure 2.

**Table 4:** SEM paths (predicting financial anxiety).

	**Coef**.	**SE**	***p***	**Path**
Job insecurity	0.43	0.06	0.00	Direct
Work related flow	−0.09	0.01	0.00	Direct

*Covariates: age, gender, marital status, employment status, income, self-rated health, sector of employment, and COVID*.

The association between job insecurity and financial anxiety was positive and statistically significant (β = 0.37, *p* < 0.001), the first hypothesis was then accepted, meaning that there was sufficient statistical evidence at α_0.05_ that job insecurity was significantly related to financial anxiety. A negative relationship was found between work related flow and financial anxiety (β = −0.09; *p* < 0.05). The second hypothesis was accepted. All these paths were significant while controlling for gender, age, marital status, employment status, income, sector of employment, self-rated health, and COVID infection status. The validity of the SEM model was confirmed by the favorable model fit indices in Table 5.

**Table 5:** SEM model goodness-of-fit.

**Measure**	**Estimate**	**Threshold**	**Interpretation**
DF	357		
CFI	0.91	>0.90	Excellent
SRMR	0.06	<0.08	Excellent
RMSEA	0.06	<0.08	Excellent
PClose	1	>0.05	Excellent

The Comparative Fit Index was CFI = 0.91, which was greater than the prescribed threshold of 0.90. The Standardized Root Mean Residual, SRMR = 0.06, was less than the prescribed maximum threshold of 0.08. Lastly, the Root Mean Square Error of Approximation (RMSEA) was 0.06, which was <0.08, the prescribed maximum. As all the model fit measures were within their prescribed thresholds, the tested model was validated (Hair et al., 2014; Fuller et al., 2016; Kline, 2016).

Prior to mediation analysis, Baron and Kenny (1986) recommended four steps that have to be followed: first, the independent variable should be significantly related to the dependent variable. Second, the independent variable should be significantly related to the hypothesized mediation variable. Third, the hypothesized mediating variable should be related to the dependent variable. Fourth, a reduction in the effect of the independent variable on the dependent variable must be observed when controlling for the mediation variable.

The results showed that the association between job insecurity and financial anxiety was statistically significant (β = 0.43, *p* < 0.001). Secondly, job insecurity was significantly related to work-related flow (β = −0.36, *p* < 0.001). Thirdly, work-related flow was significantly related to financial anxiety (β = −0.14, *p* < 0.001). Fourthly, a reduction in the effect of job insecurity on financial anxiety was observed when controlling for work-related flow (β = 0.40, *p* < 0.05). Therefore, work-related flow mediated the relationship between job insecurity and financial anxiety. The third hypothesis was also accepted as there was enough evidence against the null hypothesis. The average causal mediation effects (ACME), the average direct effects, and the proportion mediated were calculated using the “mediation” package (Tingley et al., 2014) and the results are presented in Table 6.

**Table 6:** Results of the mediation role of work-related flow.

**Effect**	**Estimate**	**95% CI lower**	**95% CI upper**	***p*-value**
ACME	0.02	0.01	0.05	<0.001
ADE	0.40	0.33	0.47	<0.001
Total effect	0.43	0.37	0.50	<0.001
Prop. Mediated	0.06	0.03	0.11	<0.001

## Discussion

Employees are experiencing increased perceptions of job insecurity due to COVID-19, which has resulted in decline of overall well-being. The present study sought to investigate the relationships between Job Insecurity (JINS), Work-Related Flow (WOLF), and Financial Anxiety (FAS) in employees of private and government sectors. It was hypothesized that a positive relationship would be found between job insecurity and financial anxiety, a negative association would be found between work-related flow and financial anxiety, and finally that a mediation role of work-related flow would be found in the relationship of job insecurity with financial anxiety. The findings supported our first hypothesis, as the results indicated a positive relationship present between JINS and FAS. The second hypothesis was also supported as a negative relationship was found between work-related flow and financial anxiety. A significant mediation of work-related flow was found in the relationship between job insecurity and financial anxiety which supported our third hypothesis. All these associations were significant while controlling confounding variables.

The results are consistent with prior studies that found that job insecurity was associated with financial anxiety (Choi et al., 2020; Sarwar et al., 2021). Widespread job insecurity increases the risk of mental health issues (Forbes and Krueger, 2019). When the Great Recession occurred, job-related issues were associated with significant anxiety and depressive symptoms for 3–4 years after the recession ended at least (Forbes and Krueger, 2019). A study by Burgard et al. (2012) indicated that employees experienced the features of depression and anxiety with job insecurity. These findings are considered especially relevant to the current COVID-19 pandemic, as many employees have experienced significant job insecurity because of the pandemic. Increased perceptions of job insecurity due to COVID-19 were indirectly related to increased symptoms of anxiety through financial concerns (Wilson et al., 2020).

Greenhalgh and Rosenblatt (2010) has called out the need to investigate the associations between job insecurity and its consequences through mediating variables. This study has found a mediating role of work-related flow in the relationship between job insecurity and financial anxiety. Job insecurity due to COVID-19 impacts on the work-related flow of employees, which in turn impacts on the financial anxiety. According to Locke (1978), employees who experience job insecurity are less motivated toward their task/activity. And those employees with high-level work-related flow have great motivation for their work. So, the results can be explained by employee motivation, as it plays roles in the experience of job insecurity and also in the maintenance of work-related flow. Moreover, Doherty and Horsted (1995) explained that employee performance is only dependent on their motivation and ability to work with high flow. Those employees who are motivated and highly focused on their job receive sufficient wages not to experience financial concerns and anxiety because they do not experience job insecurity.

### Implications of the Study

Work-related flow is the least researched construct for employees in the government and private sectors. The current study will help develop an understanding of it on behalf of those employees. Currently, there is no relevant literature on the relationships between job insecurity, work-related flow, and financial anxiety, especially in Saudi Arabia. Nor is there any literature on the role of work-related flow in the relationship between job insecurity and financial anxiety. As a result, this study will be helpful in understanding the mediating role of work-related flow for job insecurity and financial anxiety. This present study might have organizational significance as well. Organizational psychologists can use it to design policies for employees to lessen employee insecurity by maintaining sound levels of work-related flow which might reduce their financial anxiety. This study will enable theorists to understand work in greater detail. Policymakers can try to ensure job security and make friendly policies, so their employees don't experience financial anxiety.

### Limitations

There are some limitations of this research in that it has been conducted during COVID-19 conditions. Due to the rare and unprecedented effects of COVID-19, several studies indicate that individuals have started experiencing mental health issues, like anxiety, stress, or depression. The present research focuses on employee JINS, WOLF, and FAS. But in the current scenario, there might be other factors negatively impacting the individual's WOLF, such as social distancing or needing to adapt to new situations. The study had a cross-sectional design, no causal relationships can be inferred. Moreover, future studies should explore the details and nature of work for those employees working from home during COVID-19.

## Conclusion

During the COVID-19 pandemic, there have been huge unemployment levels worldwide. Vast numbers of people have lost their jobs and experienced financial pressure, which has consequently impacted the job insecurity, work-related flow, and financial anxiety of many others as well. This study has explored how greater job insecurity among employees due to COVID 19 is significantly linked with financial anxiety. It also explores the effect of work-related flow on job insecurity and financial anxiety. Further research is needed to understand the consequences of job insecurity and financial anxiety in depth, thereby investigating the role of work-related flow.

## Data Availability Statement

The original contributions presented in the study are included in the article/supplementary material, further inquiries can be directed to the corresponding author/s.

## Ethics Statement

Ethical approval for conducting this study was obtained from Umm Al-Qura University in Saudi Arabia. All procedures performed in this study were in accordance with the ethical standards of the responsible committee on human experimentation (institutional and national) and with the 1964 Helsinki Declaration and its later amendments or comparable ethical standards. The participants provided their written informed consent to participate in this study.

## Author Contributions

All authors listed have made a substantial, direct and intellectual contribution to the work, and approved it for publication.

## Conflict of Interest

The authors declare that the research was conducted in the absence of any commercial or financial relationships that could be construed as a potential conflict of interest.
